# Catheter-Dependent Patients Undergoing GreenLight Laser Photoselective Vaporisation of the Prostate: A Retrospective Observational Study of Postoperative Catheter-Free Outcomes

**DOI:** 10.7759/cureus.95650

**Published:** 2025-10-29

**Authors:** A B Azharul Islam, Maisha Zaman Poushi

**Affiliations:** 1 Urology, Countess of Chester Hospital, Chester, GBR; 2 Surgery, Dhaka Medical College & Hospital, Dhaka, BGD

**Keywords:** benign prostatic hyperplasia (bph), catheter dependence, greenlight laser photoselective vaporisation of the prostate (gll.pvp), trial without catheter (twoc), urinary retention

## Abstract

Objective: This study aimed to evaluate first-time trial without catheter (TWOC) outcomes and postoperative urinary retention rates during short-term follow-up at three to six months in men with long-term catheterisation (LTC) or intermittent self-catheterisation (ISC) undergoing GreenLight laser photoselective vaporisation of the prostate (GLL.PVP).

Methods: This retrospective observational study included 20 consecutive men with benign prostatic hyperplasia (BPH) who were dependent on LTC or ISC and underwent GLL.PVP between May 2023 and July 2024 at a tertiary centre in the United Kingdom. During this period, 50 GLL.PVP procedures were performed, and these 20 cases represented all eligible catheter-dependent patients who met the inclusion criteria. Data were collected from clinical health records, including postoperative documentation, lower urinary tract symptoms (LUTS) clinic notes, TWOC clinic assessments, and emergency department presentations with urinary retention. Parameters analysed included patient age, prostate volume, catheter status, high-risk factors, and objective voiding parameters such as post-void residual urine volume (PVR) and maximum urinary flow rate (Qmax). The primary endpoint was first-time TWOC success, and the secondary endpoint was catheter-free status at three to six months.

Results: The mean patient age was 72.8 ± 8.6 years, and the mean prostate volume was 72.5 ± 36.3 mL. Prior to surgery, 18 patients (90%) were managed with LTC and two (10%) with ISC. Nine patients (45%) were identified as high-risk due to anticoagulation therapy, prostate volume greater than 100 mL, or recurrent urinary retention. First-time TWOC was successful in 15 patients (75%), while five patients (25%) failed. At three to six months, 16 patients (80%) remained catheter-free, whereas four patients (20%) experienced recurrent urinary retention requiring re-catheterisation. Among patients who achieved catheter-free status postoperatively, the average PVR was 95 mL, and the average maximum urinary flow rate (Qmax) was 12.8 mL/s.

Conclusion: GLL.PVP appears to be a safe and feasible intervention for catheter-dependent men with BPH, demonstrating encouraging short-term outcomes with a high rate of successful first-time TWOC and sustained catheter-free status. These findings provide preliminary evidence supporting the potential role of GLL.PVP as a minimally invasive option for restoring spontaneous voiding in men with chronic catheter dependence.

## Introduction

Benign prostatic hyperplasia (BPH) is one of the most common conditions affecting older men and is a frequent cause of lower urinary tract symptoms (LUTS) and urinary retention [1]. In men with chronic urinary retention, management often involves long-term catheterisation (LTC) or intermittent self-catheterisation (ISC). Although effective for bladder decompression, chronic catheterisation is associated with recurrent infection, stone formation, discomfort, and reduced quality of life [2].

Surgical treatment is often required to restore voiding function. Transurethral resection of the prostate (TURP) has long been considered the standard intervention; however, it is associated with significant morbidity, bleeding risk, and perioperative complications, particularly in elderly patients or those on anticoagulant therapy [3,4]. Minimally invasive techniques, including GreenLight laser photoselective vaporisation of the prostate (GLL.PVP), have been developed as alternatives, offering excellent haemostasis, shorter hospitalisation, and wider applicability in high-risk patients [5].

Clinical studies have demonstrated that GLL.PVP provides functional outcomes comparable to TURP, including improvements in maximum urinary flow rate (Qmax), post-void residual urine volume (PVR), and symptom scores [6-9]. The GOLIATH trial confirmed the non-inferiority of GLL-PVP compared with TURP, with reduced perioperative complications and faster recovery [8]. Moreover, studies focusing on men presenting with urinary retention have shown that the majority achieve catheter-free status following GLL-PVP [10-12].

Men dependent on LTC or ISC before surgery remain underrepresented in the literature. Chronic catheterisation and prolonged urinary retention may impair detrusor function, potentially affecting the likelihood of successful trial without catheter (TWOC) after surgical intervention. Further evaluation of outcomes in this subgroup is needed to clarify postoperative recovery and catheter-free success.

Building on previous research examining TWOC outcomes [13] and postoperative urinary retention following GLL.PVP [14], this study aimed to evaluate first-time TWOC outcomes and short-term catheter-free status in men with LTC or ISC undergoing GLL.PVP. The study provides exploratory evidence intended to guide future studies in this specific patient group.

## Materials and methods

Study design

This retrospective observational study included all consecutive catheter-dependent men who underwent GLL.PVP for BPH at a tertiary centre in the United Kingdom between May 2023 and July 2024. During this period, 50 GLL.PVP procedures were performed, of which 20 patients (40%) were catheter-dependent preoperatively, managed with either LTC or ISC. These 20 cases represented the entire eligible cohort.

Inclusion and exclusion criteria

Inclusion criteria were patients with BPH, dependence on LTC or ISC before surgery, and completion of GLL.PVP within the study period. Exclusion criteria were known or suspected prostate cancer, neurogenic bladder dysfunction, history of previous prostate surgery, incomplete clinical documentation, or loss to follow-up within three months after surgery.

Surgical technique and catheter protocol

Procedures were performed using the GreenLight laser system (Boston Scientific, Marlborough, MA, USA). Vaporisation was carried out using a sweeping motion to create a wide prostatic channel from the bladder neck to the verumontanum, maintaining haemostasis through laser coagulation. A 20 to 24 Fr three-way Foley catheter was inserted postoperatively with continuous saline irrigation, typically removed after 24-48 hours once urine was clear. A supervised TWOC was conducted in the postoperative surgical ward and TWOC clinic. Failed TWOC cases were re-catheterised and reassessed later.

Data collection and follow-up

Data were retrieved from electronic health records, including postoperative documentation, the LUTS clinic, the TWOC clinic, and the emergency department documentation. Variables included age, prostate volume, catheter status (LTC/ISC), high-risk features (anticoagulation, prostate volume >100 mL, recurrent retention), and voiding parameters PVR and Qmax. Outcomes were assessed at short-term follow-up (three to six months). Catheter-free status was defined as sustained spontaneous voiding without further catheterisation.

Outcomes and analysis

The primary outcome was first-time TWOC success (voiding without re-catheterisation within 24 to 48 hours). The secondary outcome was catheter-free status at short-term follow-up at 3-6 months. Continuous data were presented as mean ± standard deviation (SD) and categorical data as counts and percentages. Due to the small sample size, the analysis was descriptive rather than inferential.

## Results

Twenty patients met the inclusion criteria. The mean age was 72.8 ± 8.6 years, and the mean prostate volume was 72.5 ± 36.3 cc. Eighteen patients (90%) were managed with LTC, and two (10%) with ISC prior to surgery. Nine patients (45%) had one or more high-risk features. The demographic and preoperative characteristics of the patients are presented in Table 1.

**Table 1:** Patient Demographics and Preoperative Characteristics LTC: Long-term catheterisation; ISC: Intermittent self-catheterisation

Variables	Mean ± Standard Deviation or n (%)
Continuous variables	
Age (Years)	72.8 ± 8.6
Prostate Volume (cc)	72.5 ± 36.3
Pre-operative catheter type	
LTC	18 (90%)
ISC	2 (10%)
High-risk features	Yes = 9 (45%) ; No = 11 (55%)
Preoperative medical management	Yes = 16 (80%) No = 4 (20%)

**Figure 1:**
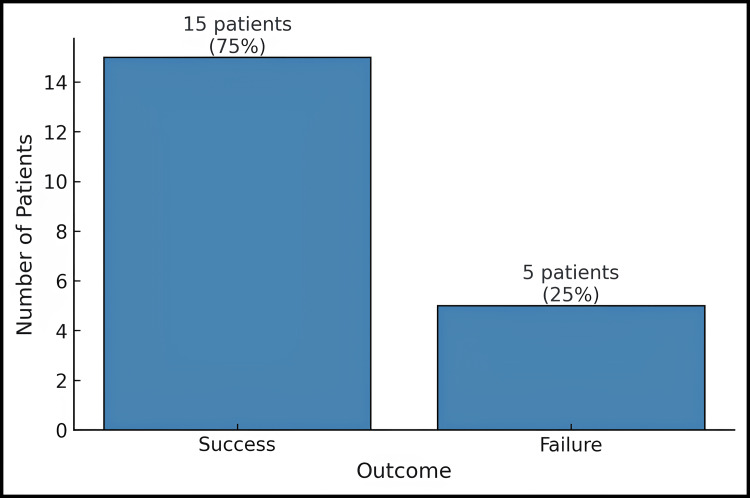
First-Time Successful TWOC Outcome TWOC: Trial without catheter

Fifteen patients (75%) achieved successful first-time TWOC, while five (25%) failed and required continued catheterisation. Successful TWOC was defined as spontaneous voiding without re-catheterisation within 24 hours of catheter removal (Figure 1).

**Figure 2:**
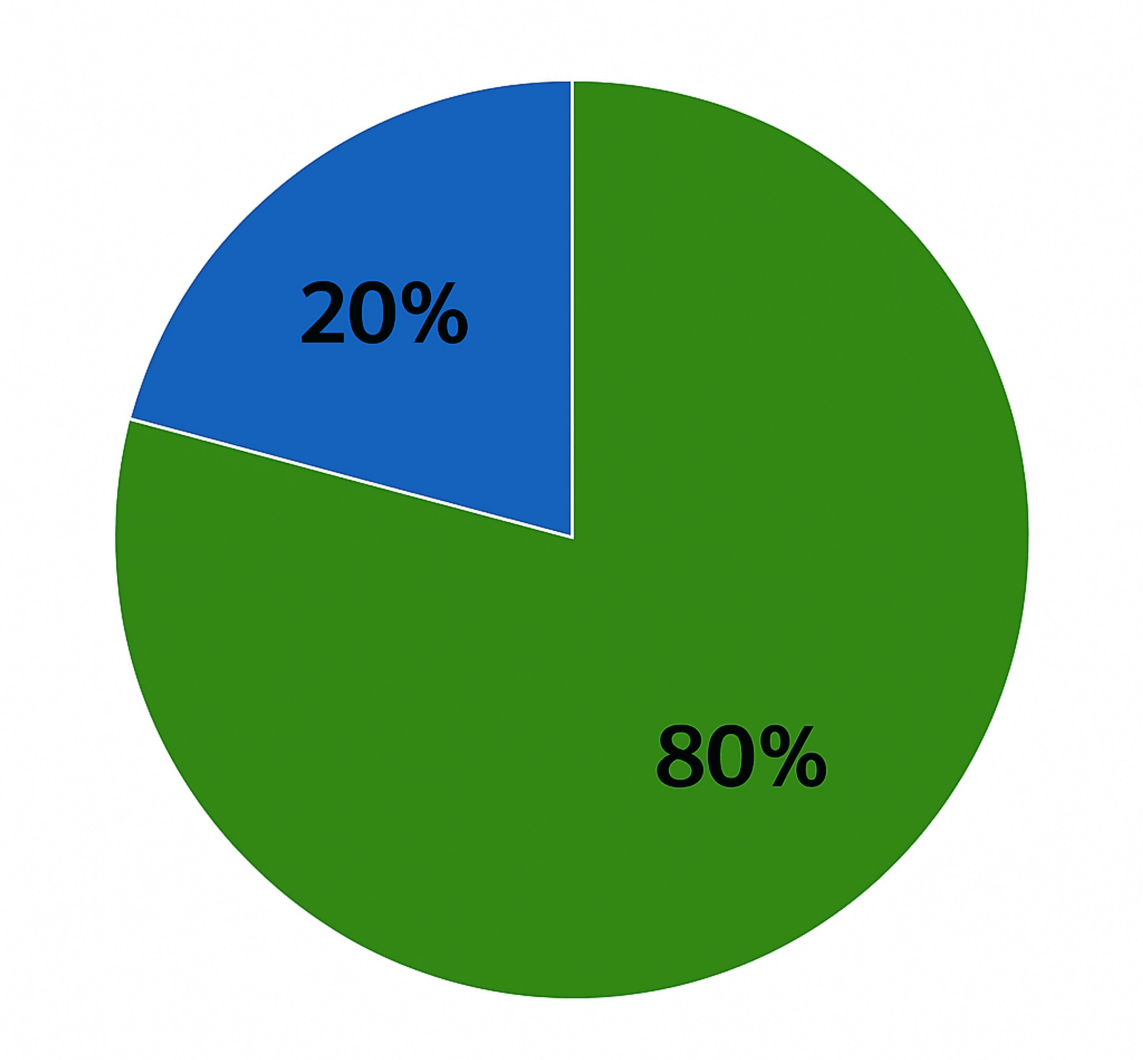
Postoperative Urinary Retention After GreenLight Laser Photoselective Vaporisation of the Prostate (GLL.PVP) in Patients at 3–6 Month Follow-Up Green colour denotes no urinary retention (80%) and blue colour denotes urinary retention (20%)

At 3-6 month follow-up, 16 patients (80%) remained catheter-free, while four patients (20%) developed urinary retention necessitating catheterisation (Figure 2).

Voiding parameters

Postoperative voiding studies demonstrated functional improvement. The mean postoperative PVR was 95 mL, and the mean postoperative Qmax was 12.8 mL/s.

## Discussion

In this study, men dependent on LTC or ISC achieved a 75% first-time TWOC success rate and 85% catheter-free status at 3-6 months following GLL-PVP. These findings are consistent with prior reports, where most patients with urinary retention regained spontaneous voiding after laser prostatectomy [10-12].

Our first-time TWOC success rate is slightly lower than that of some published cohorts. This may be explained by the high proportion of men with LTC, in whom detrusor underactivity is more prevalent. Reduced bladder contractility is a recognised predictor of TWOC failure following surgery [15,16]. Nevertheless, most patients in our series achieved durable catheter-free outcomes, indicating that GLL-PVP is effective even in this challenging population.

Nearly half of our cohort had high-risk features such as large prostates or anticoagulation therapy. No major perioperative complications such as bleeding, transfusion requirement, urinary tract infection, or clot retention were observed, and minor transient haematuria and dysuria resolved conservatively. Prior studies confirm that GLL-PVP can be performed safely in such patients, with significantly lower bleeding risk compared to TURP [17]. This aligns with our findings, where perioperative safety was maintained.

Compared with TURP, GLL-PVP has shown non-inferior long-term efficacy, with similar improvements in IPSS, Qmax, and PVR, but with reduced morbidity and transfusion rates [8,9,18]. Our data support these observations, showing meaningful improvements in voiding function and a high rate of sustained catheter-free status.

The findings from this study offer preliminary insights into the outcomes of GLL.PVP in men with chronic catheter dependence. The observed TWOC success and short-term catheter-free rates suggest that GLL.PVP may provide a feasible treatment approach in this challenging patient group. These observations are in keeping with previous studies evaluating TWOC success [13] and postoperative urinary retention [14] following GLL.PVP and contribute to the growing body of hypothesis-generating evidence supporting further prospective research with larger cohorts and extended follow-up.

Limitations of the study

This retrospective observational study is subject to several limitations. Its design inherently limits data quality and introduces potential bias, as the analysis relied on the completeness of clinical records and could not fully control for confounding variables. The cohort size was relatively small, which restricts the strength of statistical analysis and limits generalisability; however, it reflects the focus on a narrow subgroup of men with LTC or ISC, a population infrequently examined in the context of GreenLight PVP. The absence of routine urodynamic studies precluded assessment of detrusor contractility, an important variable influencing the success of TWOC and long-term voiding function. Furthermore, follow-up was confined to three to six months, and therefore, the durability of outcomes beyond this period could not be assessed. Despite these constraints, this study provides preliminary evidence on perioperative and short-term outcomes in a clinically challenging patient group, contributing to the limited body of literature on this topic.

## Conclusions

GLL.PVP appears to be a safe and feasible treatment for catheter-dependent men with BPH, demonstrating favourable early outcomes with a high rate of successful first-time TWOC and sustained catheter-free status. These findings provide preliminary, real-world evidence supporting the use of GLL.PVP as a minimally invasive management option in this challenging patient group. Larger, prospective studies with extended follow-up are required to confirm these observations and determine long-term efficacy.

## References

[1] Roehrborn CG (2005). Benign prostatic hyperplasia: an overview. Rev Urol.

[2] Desgrandchamps F, De La Taille A, Doublet JD (2006). The management of acute urinary retention in France: a cross-sectional survey in 2618 men with benign prostatic hyperplasia. BJU Int.

[3] Rassweiler J, Teber D, Kuntz R, Hofmann R (2006). Complications of transurethral resection of the prostate (TURP)--incidence, management, and prevention. Eur Urol.

[4] Reich O, Gratzke C, Stief CG (2006). Techniques and long-term results of surgical procedures for BPH. Eur Urol.

[5] Chughtai B, Te A (2011). Photoselective vaporization of the prostate for treating benign prostatic hyperplasia. Expert Rev Med Devices.

[6] Malek RS, Kuntzman RS, Barrett DM (200012025). High power potassium-titanyl-phosphate laser vaporization prostatectomy. J Urol.

[7] Ruszat R, Wyler SF, Seitz M (2008). Comparison of potassium-titanyl-phosphate laser vaporization of the prostate and transurethral resection of the prostate: update of a prospective non-randomized two-centre study. BJU Int.

[8] Thomas JA, Tubaro A, Barber N (2016). A multicenter randomized noninferiority trial comparing Greenlight-XPS laser vaporization of the prostate and transurethral resection of the prostate for the treatment of benign prostatic obstruction: two-yr outcomes of the GOLIATH study. Eur Urol.

[9] Elmansy H, Baazeem A, Kotb A, Badawy H, Riad E, Emran A, Elhilali M (2012). Holmium laser enucleation versus photoselective vaporization for prostatic adenoma greater than 60 ml: preliminary results of a prospective, randomized clinical trial. J Urol.

[10] Pradère B, Peyronnet B, Decock A, Brichart N, Bertrand P, Bruyère F (2015). Photoselective vaporization of the prostate in men with refractory urinary retention. Urology.

[11] Woo H, Reich O, Bachmann A (2008). Outcome of Greenlight HPS 120-w laser therapy in specific patient populations: those in retention, on anticoagulants, and with large prostates (≥80 mL). Eur Urol Suppl.

[12] Elshal AM, Elkoushy MA, El-Nahas AR, Shoma AM, Nabeeh A, Carrier S, Elhilali MM (2015). GreenLight™ laser (XPS) photoselective vapo-enucleation versus holmium laser enucleation of the prostate for the treatment of symptomatic benign prostatic hyperplasia: a randomized controlled study. J Urol.

[13] Islam AB, Poushi MZ (2024). First-time successful trial without catheter (TWOC) after greenlight laser photoselective vaporization of the prostate (GLL PVP) surgery for an enlarged prostate. Cureus.

[14] Islam AB, Ellis D, Chari N, Mccomb K, Poushi MZ, Donkov I (2024). Urinary retention after Greenlight laser photoselective vaporization of the prostate (GLL.PVP) surgery for benign prostatic hyperplasia (BPH): a 3-6 month retrospective follow-up study. Cureus.

[15] Lee KH, Kuo HC (2019). Recovery of voiding efficiency and bladder function in male patients with non-neurogenic detrusor underactivity after transurethral bladder outlet surgery. Urology.

[16] Bansal A, Arora A (2017). Predictors of successful trial without catheter following acute urinary retention in benign prostatic enlargement: a single centre, multivariate analysis. Neurourol Urodyn.

[17] Ruszat R, Wyler S, Forster T (2007). Safety and effectiveness of photoselective vaporization of the prostate (PVP) in patients on ongoing oral anticoagulation. Eur Urol.

[18] Reich O, Gratzke C, Bachmann A (2008). Morbidity, mortality and early outcome of transurethral resection of the prostate: a prospective multicenter evaluation of 10,654 patients. J Urol.

